# A study on the reliability and validity of the Korean version of the parenting outcome expectancy scale for parents of elementary school students

**DOI:** 10.3389/fpsyg.2023.1165783

**Published:** 2023-08-04

**Authors:** Yoonjung Kim, Jungmin Lee, Ratchneewan Ross

**Affiliations:** ^1^College of Nursing, Konyang University, Daejeon, Republic of Korea; ^2^School of Nursing, Hallym University, Chuncheon, Republic of Korea; ^3^School of Nursing, University of Louisville, Louisville, KY, United States

**Keywords:** adolescent, communication, factor analysis, parenting, sexual health

## Abstract

**Introduction:**

Many parents do not engage in active discussions with their children about sexuality. This can contribute to negative sexual and reproductive health outcomes among youth. To foster a healthy environment for sexual activity, it is crucial for parents to provide comprehensive sex education to their children at home. This study aims to cross-culturally adapt and evaluate the psychometric properties of a Korean version of the Parenting Outcome Expectancy Scale to measure the sexual communication abilities of parents of elementary school students in South Korea.

**Method:**

The study participants were parents of elementary school students between 6 to 13 years old. We used exploratory and confirmatory factor analyses to examine the reliability and validity of the 23-item Korean version of the Parenting Outcome Expectancy Scale.

**Results:**

The study confirms the reliability and validity of the scale, comprising five factors and 22 items, for the evaluation of the outcome expectancy of communication about sex between parents and their children. Results also demonstrate that talking about sex is still a challenge for many parents.

**Discussion:**

This instrument can help parents prepare for sex communication with their children and for sexual education, potentially yielding a positive effect on children’s sexual health and parental satisfaction.

## 1. Introduction

Puberty is a critical developmental stage characterized by the onset of secondary sexual characteristics, hormonal changes, and reproductive maturation (Rhie and Lee, 2015). It marks the transition from childhood to adolescence in a person’s life and plays a significant role in shaping their physical, psychological, and social well-being (Kim and Lee, 2016). However, in recent years, there has been growing concern regarding the declining age of puberty onset, specifically the occurrence of precocious puberty, among youth (Rhie and Lee, 2015). Precocious puberty refers to the early onset of pubertal development, occurring before the normal age for a specific population (Nam and Choi, 2020). This phenomenon has become a social problem, raising questions about its potential impact on the sexual attitudes, behaviors, and overall well-being of the youth (Nam and Choi, 2020). Previous studies have indicated a link between early physical maturation and an increased likelihood of engaging in risky sexual behavior among the youth (Hong and Hwang, 2013; Cheong et al., 2015; Lee, 2017; Shin H. W. et al., 2019; Shin H. et al., 2019). Precocious puberty adolescents may experience heightened sexual curiosity and interest, which can lead to earlier sexual initiation and engagement in risky sexual activities (Hong and Hwang, 2013; Lee, 2017). The physical changes associated with precocious puberty, such as the development of secondary sexual characteristics, can influence self-perception, body image, and social interactions, potentially affecting sexual decision-making processes (Wie and Kim, 2014; Park et al., 2018). Engaging in risky sexual behavior during adolescence poses various threats and potential negative consequences (CDC, 2021). Early sexual initiation is associated with an increased risk of unintended pregnancies, sexually transmitted infections (STIs), emotional distress, and compromised mental health (Hong and Hwang, 2013; Lee, 2017). Furthermore, youth who engage in risky sexual behavior may face challenges when negotiating safe sexual practices, understanding consent, and accessing appropriate sexual health services (Hong and Hwang, 2013; Lee, 2017).

In this context, parents can play a unique and powerful role in shaping their children’s sexual attitudes and behaviors, thereby molding them into sexually healthy adults (Somersa and Anagurthia, 2013). Barriers to open discussions on reproductive health problems exist not only due to parents’ lack of age-appropriate and respectful vocabulary and skills, but also due to cultural norms and taboos related to sexual and reproductive health issues (Motsomi et al., 2016). Communication between parents and youth about sexual and reproductive health issues increases awareness of the issues and protects youth from negative consequences and sexual problems (Motsomi et al., 2016). However, studies have found that it is difficult for most Asian parents to educate their children about sensitive sexual problems and sexual health (Motsomi et al., 2016; Baku et al., 2018). There are several factors that can contribute to difficulties faced by many Asian parents when it comes to educating their children about these matters. First, Asian cultures often have conservative values and strict social norms surrounding discussions about sexuality, especially with children (Lee, 2019). Sex is considered a private and sensitive topic, and thus, there may be general discomfort or reluctance to openly address sexual matters within the family. This cultural context can create barriers to effective communication about sexual health. Second, in some Asian countries, existing formal education system may not provide comprehensive or adequate sex education (Kim et al., 2018). This places greater responsibility on parents to fill the gap, but the lack of knowledge and resources can make it difficult for them to effectively educate their children about sexual health, thus creating a weak knowledge base. Third, language and generational gaps can be barriers to effective communication between parents and children regarding sensitive sexual topics (Lee and Kim, 2017; Jo et al., 2018). Parents may struggle to choose appropriate vocabulary, or may feel embarrassed or awkward when discussing such matters. Similarly, children may feel uncomfortable initiating conversations with their parents due to cultural or language barriers. Fourth, parents may worry about the potential judgment or stigma that could arise from openly discussing sexual topics with their children (Lee, 2019). They may fear that discussing such matters could be seen as encouraging or promoting sexual activity, leading to concerns about their reputation within their community. Fifth, some parents may have limited knowledge about sexual health themselves, which can undermine their confidence in discussing the subject with their children (Lee and Kweon, 2013; Lee and Oh, 2016). They may feel ill-equipped to provide accurate information or guidance, leading to a reluctance to engage in conversations about sexual health. Lastly, in some Asian cultures, especially South Korea, there is strong emphasis on academic success, and discussions about sexuality may be seen as a distraction from educational pursuits (Jwa, 2014; Soh et al., 2014). Parents may prioritize academic achievements over addressing sexual health matters, leading to a lack of focus in this aspect of their children’s development. Those challenges may induce negative sexual and reproductive health outcomes among the youth (Somersa and Anagurthia, 2013). To create and promote a healthy climate for sexual activity in the future, parents should make every effort to provide appropriate sex education to their children at home. If conversations regarding sex-related topics take place naturally between parents and their children, children may be able to establish appropriate sexual values and cope with sexual problems effectively (Baku et al., 2018).

In this regard, parents play a key role in influencing positive changes in their children’s sexual behavior as they advance into adulthood. However, in South Korea, there are limited tools to directly measure parents’ abilities to communicate about sex to their children. Therefore, this study aimed to cross-culturally adapt and evaluate the psychometric properties of a Korean version of the Parenting Outcome Expectancy Scale (KR-POES) to measure the sexual communication abilities of parents of elementary school students in South Korea. The KR-POES aims to measure the sexual communication abilities of parents of elementary school students in South Korea, offering insights into their current skills and attitudes. Effective parent–child communication about sexual health has been linked to positive sexual health outcomes among the youth (Lee and Kim, 2017; Jo et al., 2018; Lee, 2019). A validated tool like the KR-POES can help parents receive targeted guidance and support to engage in open and constructive discussions about sex with their children. This, in turn, can contribute to improved sexual health outcomes and well-being for young people. This assessment is crucial in understanding parents’ current abilities and attitudes towards discussing sexual health topics with their children. On validation, the KR-POES can be used as an assessment tool to measure the effectiveness of interventions aimed at improving parents’ sexual communication skills over time. Longitudinal evaluation allows researchers and practitioners to track changes in parental behavior and attitudes, helping to determine the impact of interventions to enhance parent–child communication about sexual health. Furthermore, the KR-POES can help identify specific areas where parents may need more support or education regarding sexual communication. By assessing parents’ outcome expectancies related to sexual health discussions, researchers and practitioners can pinpoint areas of weakness or misconceptions that can be targeted with tailored interventions to improve parental skills and knowledge. Overall, the study on the KR-POES is important for establishing a reliable and validated instrument that can aid in assessing and improving parents’ sexual communication abilities. This has the potential to help in developing effective interventions, monitor socio-cultural changes, and ultimately enhance sexual health outcomes for young people in South Korea.

## 2. Materials and methods

### 2.1. Design

We used exploratory and confirmatory factor analyses to examine the reliability and validity of the 23-item Korean version of the POES originally developed by Dilorio et al. (2006).

### 2.2. Participants

The study participants were parents of elementary school students aged 6 to 13 years old. In the period of elementary school when puberty begins, sex has a particularly important meaning (Rhie and Lee, 2015). This is because the reproductive organs begin to mature and through cognitive development, one observes their reality with new possibilities of designing one’s own life; thus, there is both a mental and physical component (Cho and Moon, 2016). In other words, in adolescence, there is a clear physiological development of the body, and a specific sexual concept is formed accordingly; the attitude towards sex formed at this time affects adult life. Thus, in this study, parents of elementary school students between 6 to 13 years old were recruited as target subjects. The inclusion criteria were (1) parents with at least one child in elementary school and (2) parents who could communicate in Korean. The number of participants was determined based on a classic recommendation that more than 200 subjects are needed to obtain reliable factors during factor analyses. Therefore, the estimated minimum number of participants was 224, eight times the number of items; anticipating an incomplete survey rate of 20%, we recruited 250 participants for our study (DeVellis, 2017).

### 2.3. Measures

#### 2.3.1. Parenting outcome expectancy scale (POES)

Based on social cognitive theory (Bandura, 1997), the POES was developed to measure parents’ expectations of the outcomes associated with communicating with their adolescent children about sex-related topics. The original instrument was validated for reliability and validity with parents of children aged 11 to 14 years old (Dilorio et al., 2001). Considering the earlier onset of puberty and the positive effects of starting sexual communication at a younger age, KR-POES was expanded to include Korean elementary school parents, ages 6 to 13. The original version of the POES included 15 items (Dilorio et al., 2001); eight items were added later, resulting in a total of 23 items. There are three factors: the self-evaluative, emotional self-evaluative, and social components. The items are scored on a five-point Likert scale, and the scores of each item are summed, with higher scores indicating more positive outcome expectancies. In the original study, the 23-item scale was shown to have good internal consistency, with a Cronbach’s alpha of 0.83. In the 22-item scale of the current study, Cronbach’s alpha was 0.88.

### 2.4. Procedures

#### 2.4.1. Translation procedure

The authors obtained permission to use the POES from the original authors before the study. Thereafter, KR-POES was translated and adapted to measure parental expectations of outcomes related to communicating with their adolescent children about sex-related topics. The instrument translation process adhered to the World Health Organization (2015) guidelines for instrument translation and application. First, two bilingual translators translated the instrument from English to Korean. The authors then discussed the translation’s vocabulary, clarity, and whether cultural differences necessitated any modifications. A second independent translator who was fluent in English and Korean then completed the back translation. The authors then reviewed both versions to identify and resolve inconsistencies between the original English and translated Korean versions. Two additional independent translators then repeated the procedure. The committee of authors then reexamined the social and cultural aspects of the forward and back translations. After reaching a consensus, minor modifications were conducted to account for improved grammatical clarity and item parity. The instrument was completely based on the suggestions of the committee members. A total of 23 items were included in the final version of the KR-POES. Finally, we recruited five parents of elementary schools in South Korea to establish face validity. All students stated that the tool was easy to understand and had good flow.

#### 2.4.2. Content validity

Three experts in sexual health and two in child health nursing evaluated the forward-backward translated questionnaire to confirm its content validity. At this stage, pre-testing and cognitive interviewing were conducted by experts for clarity, flow, and to address social and cultural considerations. An item-level content validity index was used, in which a score greater than 0.80 indicated content validity. In this study, all 23 items were rated 0.80 or higher; therefore, all items were retained for factor analysis.

#### 2.4.3. Data collection

Prior to the study procedures, the authors obtained approval from the institutional review board of H University (HIRB-2022-045). Data were collected in August and September 2022. Participants were recruited using convenience sampling; we posted advertisements regarding recruitment of participants for our study on social media networking services and recruited respondents who met our selection criteria from among those who viewed the posts. Data were collected via an online survey form. Participation was voluntary, and withdrawal was possible at any time.

#### 2.4.4. Validity testing

##### 2.4.4.1. Exploratory factor analysis (EFA)

An EFA with varimax rotation was performed below oblique rotations. Data factorability was assessed using the Kaiser-Meyer-Olkin (KMO) measure of sampling adequacy (which should ideally be ≥0.80). To evaluate factorability in terms of the magnitude of intercorrelations and sampling adequacy, Bartlett’s test of sphericity (which should be significant at *p* < 0.05) was also performed. Subsequently, a screen test of eigenvalues was used against these factors. Thereafter, the proportion of variance accounted for by a factor was measured using the cumulative proportion (%) of the variance. Finally, we checked the pattern matrix, which contained the factor loading for each factor of primary interest.

##### 2.4.4.2. Confirmatory factor analysis (CFA)

CFA was performed on the two extracted factors using EFA. All items were loaded onto factors (>0.30 indicates fair loadings). The popular indices of model fit included the model chi-square, *df* and its *p*-value, Steiger-Lind root mean square error of approximation and its 90% CI (between 0.06–0.10 indicates adequate model fit), and Bentler comparative fit index (>0.95 indicates good model fit).

##### 2.4.4.3. Criterion validity

In this study, the KR-POES was examined in relation to the Brief Parenting Self-Efficacy Scale (BPSES) and Parent-Adolescent Communication Scale (PACS) because it makes sense theoretically that the outcomes of the parents’ communication with their child about sex-related topics should be associated with the parents’ self-efficacy and ability to communicate with their child in general.

Specifically, we selected an instrument of criterion validity, considering that POES is based on social cognitive theory. Bandura’s (1997) social cognitive theory categorizes outcome expectancy into three distinct types: self-evaluative, social, and physical. Self-evaluative outcome expectancy pertains to individual responses. Given that self-efficacy is a pivotal concept within Social Cognitive Theory and is responsive to outcome expectations, we considered parental self-efficacy as one of the metrics for criterion validity. Furthermore, high positive outcome expectations resulting from parental sexual communication are strongly associated with the actual enactment of behavior (Bandura, 1997). Consequently, we sought to measure criterion validity utilizing the Parent-Adolescent Communication Scale (PACS).

###### 2.4.4.3.1. Brief parenting self-efficacy scale

The BPSES was developed by Woolgar et al. (2012) to measure parental self-efficacy (Woolgar et al., 2012; Selwyn et al., 2016). The instrument comprises of five items rated on a five-point Likert scale, with a higher score indicating a higher level of parental self-efficacy. The Cronbach’s alpha was 0.75 in the original study (Selwyn et al., 2016) and 0.78 in the current study.

###### 2.4.4.3.2. Parent-adolescent communication scale

The PACS is an instrument developed by Barnes and Olson (1982) and cross-culturally adapted by Min (1992). It includes 20 open-and problem-communication items rated on a five-point Likert scale. Items 2, 4, 5, 10, 11, 12, 15, 18, 19, and 20 are reverse scored. Higher scores indicate better communication between the parent and child. The Cronbach’s alpha in the original study ranged from 0.78 to 0.87 (Barnes and Olson, 1982); for this study, it was 0.70 and 0.85 for problem and open communication, respectively.

##### 2.4.4.4. Internal consistency reliability

To examine the reliability of the KR-POES, item-total correlations were generated along with a Cronbach’s alpha to show internal consistency.

### 2.5. Data analysis

The SPSS Amos v. 25.0 (IBM Corp., Armonk, NY, United States) was used for statistical analysis. Descriptive statistics were used to summarize the participants’ demographic characteristics and for item and Cronbach alpha analyses. Construct validity was determined through EFA and CFA along with the correlations between the KR-POES and BPSES, and between the KR-POES and PACS.

## 3. Results

### 3.1. Participants’ general characteristics

Two hundred and fifty participants with children enrolled at an elementary school were recruited for this study (mean age = 42.36 ± 4.14). The youngest parent was aged 27 years, and the oldest, 58. Among these, the proportion of fathers was 52.8% (*n* = 132) and mothers, 47.2% (*n* = 118). The majority of these participants were college graduates (79.2%, *n* = 198). The economic level was largely reported to be average (85.2%, *n* = 213). Additionally, 60% had two children, and approximately 30% had only one child (Table 1).

**Table 1:** General characteristics (*N* = 250).

		*N* (%)	Mean ± SD (min–max)
Gender	Male	132 (52.8)	
	Female	118 (47.2)	
Age (years)	Under 39	59 (23.6)	42.36 ± 4.14 (27–58)
	Over 40	191 (76.4)	
Final education status	High school graduate	11 (4.4)	
	College graduate	198 (79.2)	
	Graduate school graduate	41 (16.4)	
Economic level	Low	20 (8.0)	
	Average	213 (85.2)	
	High	17 (6.8)	
Children (number)	One	70 (28.0)	
	Two	154 (61.6)	
	Three	22 (8.8)	
	Four	4 (1.6)	
Sexual tolerance	Conservative	66 (26.4)	
	Average	147 (58.8)	
	Open	37 (14.8)	
Usual interest in sexual issues or topics	Never interested to somewhat not interested	17 (6.8)	
	Neutral	89 (35.6)	
	Partly interested	116 (46.4)	
	Very interested	28 (11.2)	
Parents’ influence on children’s sexual attitudes	Not at all to somewhat influential	5 (2.0)	
	Neutral	18 (7.2)	
	Somewhat influential	170 (68.0)	
	Very influential	57 (22.8)	
Has your child ever asked you about sexual issues or topics?	Yes	114 (45.6)	
	No	136 (54.4)	
Have you ever received education on how to teach and/or explain sexual issues to your child?	Yes	78 (31.2)	
	No	172 (68.8)	
How often do you talk with your child about sexual issues or topics?	Never	81 (32.4)	
	Once a year	104 (41.6)	
	Once a week or month	65 (26.0)	
What do you do if you have to explain sexual issues to your child?	Avoid answering	5 (2.0)	
	Talk indirectly	126 (50.4)	
	Talk directly but only briefly explain the issue	53 (21.2)	
	Explain the issue in detail	66 (26.4)	
What level of knowledge about sex do you have to answer your child’s questions?	Lacking to somewhat lacking	137 (54.8)	
	Average	80 (32.0)	
	Very knowledgeable	33 (13.2)	
Do you find it difficult to talk about sexual issues or topics with your child?	Have difficulties	195 (78.0)	
	Not at all	55 (22.0)	
If yes, why do you find it difficult to talk about sexual issues or topics with your child? (*n* = 195)	Do not know what to say or how	74 (29.6)	
	Lack of materials and resources	28 (11.2)	
	Feel embarrassed and awkward	64 (25.6)	
	Concerns about adverse effects	29 (11.6)	

More than half of the parents (58.8%, *n* = 147) responded that they had average sexual tolerance, implying that they believed that they had an average ability to make autonomous decisions about their own sexual life within the boundaries of personal and social ethics. Almost a quarter of the parents (26.4%, *n* = 66) indicated that their sexual tolerance was conservative. Nearly half of the participants (46.4%, *n* = 116) indicated that they had a partial interest in sexual issues or topics, followed by a neutral interest (35.6%, *n* = 89). Furthermore, 7 out of 10 participants (68.0%, *n* = 170) believed that parents somewhat influenced their children’s sexual attitudes, while 22.8% indicated that parents were very influential in this context (*n* = 57).

Approximately four-fifths of participants (68.8%, *n* = 172) indicated that they received education on how to teach and/or explain sexual issues or topics to their children and that about half of their children (45.6%, *n* = 114) had asked about sexual issues or topics. However, only a quarter of the children (26.0%, *n* = 65) asked their parents about sexual issues or topics once a week or month; however, 41.6% (*n* = 104) of the parents talked with their children about sexual issues or topics once a year, and 32.4% (*n* = 81) of parents never received any questions from their children regarding this topic. If they faced a situation related to these issues that required an explanation, 7 out of 10 parents (71.6%, *n* = 179) responded that they would give a brief explanation in an indirect or direct manner. More than half of the parents (54.8%, *n* = 137) reported that their level of knowledge on sex was somewhat extremely lacking, and nearly 80% (78.0%, *n* = 195) felt that it was difficult to talk about sexual issues or topics with their child. Those who had trouble explaining these issues to their children reported that it was mostly because they did not know what or how to address and/or explain it (29.6%, *n* = 74) and because they felt embarrassed and awkward discussing the topic with their children (25.6%, *n* = 64).

### 3.2. Validity and reliability testing of The KR-POES

#### 3.2.1. Skewness and kurtosis

The normality of the distributions was supported by skewness and kurtosis coefficients. No items exceeded the absolute value of skewness and kurtosis of two and seven, respectively, indicating that all items met the appropriate requirements.

#### 3.2.2. Item-to-total correlations

The item-to-total correlations were measured to evaluate internal consistency (Table 2). Item 8 showed a lower value than 0.30; when the item was removed, the internal consistency increased significantly. Therefore, after an in-depth discussion, we decided to remove item 8 from the KR-POES. Although items 11 and 22 were at the borderline of the cut-off score, we decided to retain them because they did not significantly affect internal consistency.

**Table 2:** Item-to-total correlations (*N* = 250).

Items (If I talk to my child about sexual topics…)	Item-total correlation	Alpha if item deleted
I will be proud.	0.55	0.869
I will feel like a responsible parent.	0.64	0.868
I will feel that I have done the right thing.	0.61	0.869
I will be embarrassed.*	0.50	0.871
I will turn my attention to another topic.*	0.52	0.870
My child will listen carefully.	0.44	0.873
I will feel comfortable.	0.47	0.872
My child will do whatever they want to do. *	0.22	0.879
I will feel ashamed.*	0.43	0.873
I think it will help my child to some extent.	0.53	0.871
My child will be less likely to engage in sexual intercourse as a teenager.	0.29	0.877
It will be unpleasant (feeling bad).*	0.49	0.871
It will make it less likely that my child will become pregnant or get their partner pregnant.	0.49	0.871
I can easily talk about sexual issues.	0.50	0.871
I will feel relieved.	0.49	0.871
My child will be ashamed (embarrassed).*	0.44	0.873
My child will not want to talk to me.*	0.55	0.869
I would think that I did what parents are supposed to do.	0.48	0.872
My child will remember the things that I tell them when they are older.	0.47	0.872
My child will appreciate the way I try to convey knowledge.	0.35	0.875
My child will make me uncomfortable while talking.*	0.50	0.871
My child will be able to resist pressure to have sex from their peers (sympathetic pressure: pressure from the peer group to do the same thing).	0.29	0.877
My child will know what I think of teens having sex.	0.39	0.874

* Reverse scored.

#### 3.2.3. Exploratory factor analysis with Varimax rotation

An EFA with Varimax rotation on the items of the cross-culturally adapted KR-POES was performed (Table 3) and five factors were identified. The KMO sample fit measure and Bartlett sphericity verification were the same for both statistical results (KMO = 0.869, *χ^2^* = 2250.833, *df* = 231, *p* < 0.001), indicating that this model was suitable for factor analysis.

**Table 3:** EFA with Varimax rotation (*N* = 250).

No. (original no.)	Items (If I talk to my child about sexual topics…)	Factor 1	Factor 2	Factor 3	Factor 4	Factor 5
KR-POES 1 (2)	I will feel like a responsible parent.	0.667				
KR-POES 2 (3)	I will feel that I have done the right thing.	0.657				
KR-POES 3 (10)	I think it will help my child to some extent.	0.591				
KR-POES 4 (23)	My child will know what I think of teens having sex.	0.543				
KR-POES 5 (18)	I would think that I did what parents are supposed to do.	0.532				
KR-POES 6 (19)	My child will remember the things that I tell them when they are older.	0.472				
KR-POES 7 (6)	My child will listen carefully.	0.381				
KR-POES 8 (7)	I will feel comfortable.		0.703			
KR-POES 9 (1)	I will be proud.		0.629			
KR-POES 10 (14)	I can easily talk about sexual issues.		0.567			
KR-POES 11 (15)	I will feel relieved.		0.466			
KR-POES 12 (20)	My child will appreciate the way I try to convey knowledge.		0.438			
KR-POES 13 (17)	My child will not want to talk to me.*			0.853		
KR-POES 14 (16)	My child will be ashamed (embarrassed). *			0.600		
KR-POES 15 (21)	My child will make me uncomfortable while talking.*			0.583		
KR-POES 16 (12)	It will be unpleasant (feeling bad).*			0.515		
KR-POES 17 (4)	I will be embarrassed.*				0.778	
KR-POES 18 (5)	I will turn my attention to another topic.*				0.726	
KR-POES 19 (9)	I will feel ashamed.*				0.569	
KR-POES 20 (11)	My child will be less likely to engage in sexual intercourse as a teenager.					0.669
KR-POES 21 (13)	It will make it less likely that my child will become pregnant or get their partner pregnant.					0.486
KR-POES 22 (22)	My child will be able to resist pressure from their peers to have sex.					0.392
Eigenvalue		6.573	3.124	1.450	1.237	1.022
Total variance explained proportion (%)		29.876	14.199	6.593	5.622	4.646
Cumulative proportion (%)		29.876	44.075	50.668	56.290	60.936

**Figure 1:**
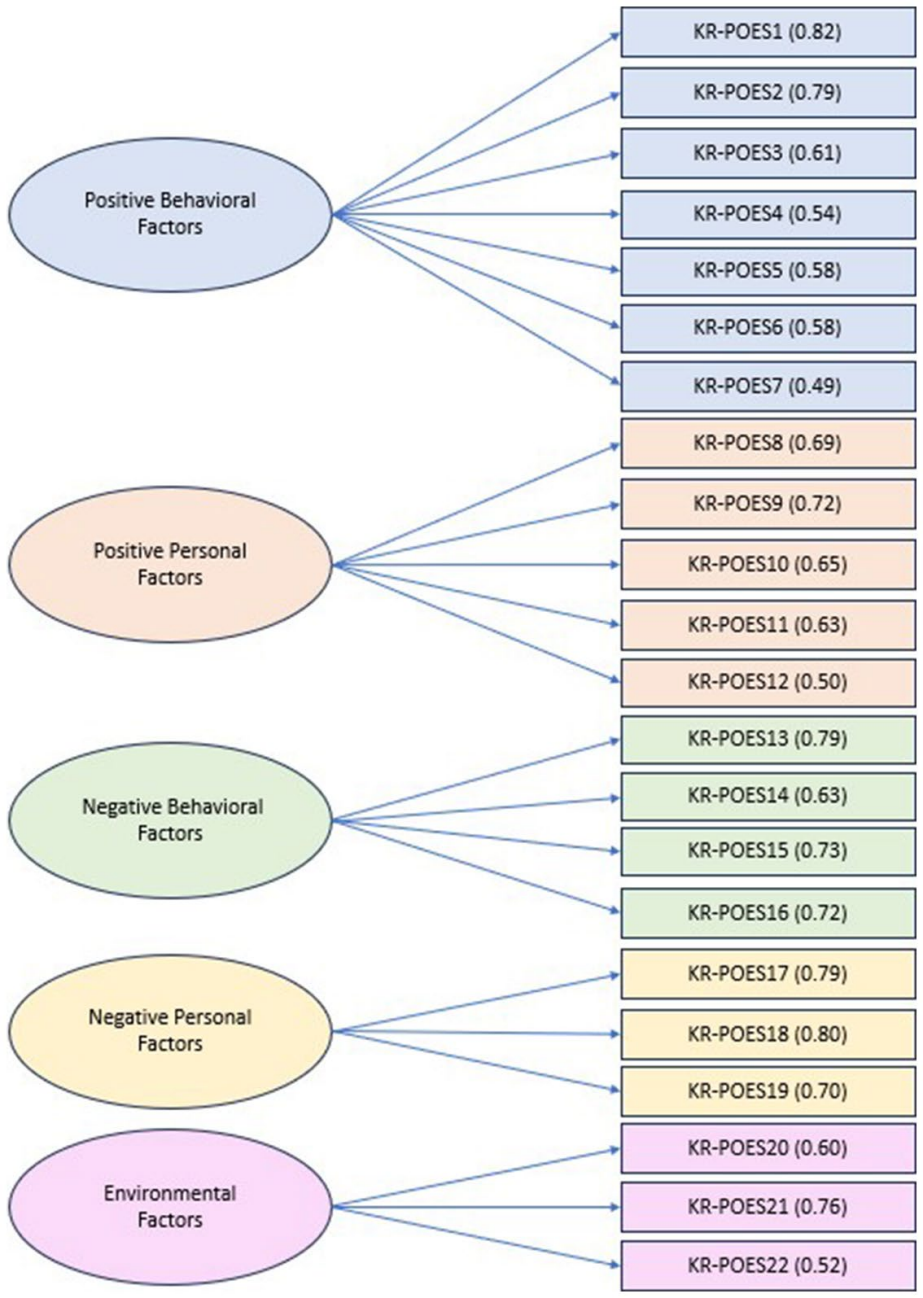
Final KR-POES with factor loading results.

The variance of each of the 22 items accounted for 60.936% of the total variance, and the factor loadings for all items were above 0.30. Additionally, there was a sharp decrease in the slope of the five factors in the scree plot. Labeling is based on Bandura’s (1997) social cognitive theory; thus, it was titled on this basis. The names of factors were labeled as “positive behavioral factors” for factor 1, “positive personal factors” for factor 2, “negative behavioral factors” for factor 3, “negative personal factors” for factor 4, and “environmental factors” for factor 5. Factor 1 comprised of seven items, with factor loadings ranging from 0.381 to 0.667. Factor 2 comprised of five items, with factor loadings ranging from 0.438 to 0.703. Factor 3 comprised of four items, with factor loadings ranging from 0.515 to 0.853. For factor four, three items were included, and the factor loading ranged from 0.569 to 0.778. All reverse-scored items were allocated to factors 3 and 4. Lastly, factor 5 comprised of three items, with factor loadings ranging from 0.392 to 0.669 (See Figure 1).

#### 3.2.4. Appropriateness of CFA

The model fit of the KR-POES was adequate and acceptable, with *χ^2^* = 355.856 (*df* = 193, *p* < 0.001), CMIN/DF = 1.844, RMSEA = 0.058 (90% CI: 0.049–0.068), RMR = 0.04, TLI = 0.91, and CFI = 0.92 (Table 4). Except for *χ^2^*, all indices satisfied the recommended level.

**Table 4:** Appropriateness of CFA (*N* = 250).

Model	*X* ^2^	CMIN/DF	RMSEA	RMR	TLI	CFI
Ideal criteria		<3	>0.05	≤0.05	>0.9	>0.9
Results	355.856 (df = 193, *p* < 0.001)	1.844	0.058 (0.049–0.068)	0.04	0.91	0.92

#### 3.2.5. Criterion validity

The correlation between the KR-POES, BPSES, and PACS showed a low to moderate positive relationship, and these correlations were all statistically significant (*r* = 0.559, *p* < 0.001; *r* = 0.185, *p* = 0.03; *r* = 0.383, *p* < 0.001, respectively) (Table 5).

**Table 5:** Correlation and Cronbach’s alpha (*N* = 250).

	1	2	3
KR-POES (1)	0.88		
BPSES (2)	0.559 (<0.001)	0.78	
PACS (3)	0.185 (0.003)	0.383 (<0.001)	0.61

#### 3.2.6. Reliability in KR-POES

Cronbach’s α for the KR-POES was 0.88 for the 22 items, showing a high reliability (Table 5). Furthermore, the five items of the BPSES had a Cronbach’s *α* of 0.78, and the 20 items of the PACS had a Cronbach’s *α* of 0.61, indicating moderate-to-high reliability.

## 4. Discussion

Results from this study showed that the KR-POES is a valid and reliable scale. Therefore, the instrument, with its culturally sensitive and psychometrically sound feature, can be used to measure the outcome expectancy of communication about sex between parents and their elementary school children.

The POES, originally framed upon Bandura’s (1997) social cognitive theory, was designed to gauge parental expectations regarding communication about sex-related topics with their children. As the theory posits, parents with a belief that such conversations yield positive outcomes are more likely to initiate them. Consequently, it is vital to discern parents’ attitudes towards these discussions, facilitating an understanding of the extent of such dialogues and the necessary preparation. Utilizing KR-POES to measure parents of Korean elementary school-aged children’s expectations about sexual communication outcomes allows us to contextualize our understanding, both locally and globally.

Contrary to the common belief that sexual communication and education should commence during adolescence, it is, in fact, pivotal to begin these discussions at a younger age, ideally during elementary school. Several parents face difficulties discussing sex with their young children, either out of a desire to protect their innocence, due to personal discomfort, or fear of judgement (Stone et al., 2013). However, initiating sexual communication at an early age is crucial. This encourages parents to believe in the positive outcomes of such communication (Astle et al., 2022), which consequently impacts children’s sexual health positively (Flores and Barroso, 2017). Elementary school-aged children express curiosity about sexuality-related issues, physiological gender differences, and childbirth (Brilleslijper-Kater and Baartman, 2000; Guder and Alabay, 2018). Discussing sexuality during childhood aids children in understanding themselves and their bodies (UNESCO, 2018), shaping safe and healthy attitudes and beliefs about sexuality (Morawska et al., 2015) that they carry forward into their lives. Hence, communication about sex and sexuality between parents and children in elementary school plays a significant role in this development, making it essential to measure parents’ expectations regarding these conversations due to their correlation with actual communication.

Communication about sex between parents and their children is an essential part of sexual health. Parents should recognize that their communication about sex is important and meaningful, which may encourage them to communicate more actively. Moreover, sexual communication between parents and children has been shown to positively affect sexual attitudes, effective contraceptive use, safe sexual behavior, and sexual health, and further reduce the spread of sexually transmitted diseases (Sieving et al., 2017; Widman et al., 2019). To improve communication about sex between early adolescent children and their parents in the future, it is necessary to first understand the current extent of sex communication using an appropriate instrument. Since sexual-health-related attitudes can be influenced by culture, we attempted to determine this level of communication in an Asian context through KR-POES.

Approximately 20 years ago, Lim and Byun (2002) found that sex education is necessary for parents, even though they find it difficult to talk to their children regarding sex. Twenty years later, the present study observed that talking about sex is still a challenge for parents. In other words, though parent–child communication about sex is important, it is not considered to be well-developed. In a previous study, Korean parents thought that it was important to provide sex education to their elementary school children, but found it difficult to apply this education in practice (Shin H. W. et al., 2019; Shin H. et al., 2019). In the present study, approximately 70% of parents had never received sex education as children. Furthermore, approximately 80% of the participants mentioned that they were uncomfortable talking about sex with their children. These results are similar to those found in China, where only 40% of participants who were parents of children similar in age to those in this study felt comfortable discussing sex with their children (Zhang and Yuan, 2023). Furthermore, in Ethiopia, only 21.3% of parents communicated with their children about sexual issues (Bekele et al., 2022). These results suggest that this is not a country-specific issue, and that the situation in each country needs to be examined by considering culture.

The original POES initially comprised of 15 items (Dilorio et al., 2001). Eight items were later added to the self-evaluative and social component to increase the scale’s validity (Dilorio et al., 2006). The updated POES thus included 23 items. After CFA, the KR-POES was finalized with 22 items. Item 8 was deleted from the original instrument (i.e., “My child will do whatever he/she wants to do”), as it did not reflect the perspective on sexual behavior in Asian cultures, which differs from that of the West. Sexual communication can manifest itself differently in different cultures (Maina et al., 2020; Toru et al., 2022)—in fact, culture is one of the barriers to communication between parents and children about sexuality (Mullis et al., 2021).

In addition to the original instrument, there are three factors in the KR-POES: the self-evaluative, emotional self-evaluative, and social components (Dilorio et al., 2001, 2006). These factors comprise of five sub-factors: (1) positive behavioral factors: social–parental role (parental responsibility); (2) positive personal factors: positive emotions; (3) negative behavioral factors: difficult or embarrassing situations; (4) negative personal factors: poor coping abilities; and (5) environmental factors: social–health outcomes. Compared with the original POES (Dilorio et al., 2001, 2006), the number of sub-factors is greater, and the composition of each item is different, which is likely due to cultural differences. In the case of Korea, an Asian country with a Confucian culture, parents still tend to intervene and control their children more actively, which is thought to be reflected in the factors of the KR-POES (Jang et al., 2016; Leung and Shek, 2018). Furthermore, the interpersonal and intrapersonal aspects are separated in the KR-POES.

In previous studies, barriers to sex communication between parents and children can be largely divided into personal, communal, and cultural barriers (Mullis et al., 2021). There were cases in which sex was regarded as taboo; in other cases, there was a generational gap (Mullis et al., 2021). This study found that the sub-factors of the KR-POES reflected the classified attributes as factors affecting communication between parents and children on sexuality. In the case of sexual communication outcome expectancy, knowledge of barriers is essential because parents must feel that they can overcome these barriers. Therefore, for parents to communicate about sex appropriately and efficiently, it is necessary to provide interventions at a suitable time by identifying factors that affect sexual communication, including culture.

We conducted a correlation analysis between the KR-POES and the BPSES and PACS to analyze its validity. The correlation between the KR-POES and the two other scales was positive and statistically significant, supporting criterion validity. Furthermore, internal consistency was evaluated to verify the reliability of the instrument. The Cronbach’s alpha coefficient of the KR-POES was 0.88, indicating good reliability. Therefore, it is judged to be an effective tool for measuring the outcome expectancy of communication about sex among Korean parents with elementary school children.

The social cognitive theory underpinning the POES indicates that preparatory measures are essential to foster sexual communication between parents and elementary school students. Having positive expectations of such communication can enhance its frequency, underscoring the importance of positive anticipation. A study in Romania revealed that parental self-efficacy and expectations of sexual communication outcomes significantly predicted the other variable, and both substantially influenced actual sexual communication (Pop and Rusu, 2019). Augmenting parental self-efficacy and outcome expectations of sexual communication could thus be an effective strategy to amplify actual communication. Hence, employing the Korean version of this instrument to assess expectations of sexual communication may enable future research to identify influencing factors, subsequently uncovering efficacious strategies that could potentially address aspects such as confidence, support, and knowledge.

Future research should use the KR-POES to examine the differences in communication between fathers and mothers. Previous studies have revealed differences in the degree of communication about sex and its effect, depending on the parent’s gender (Dilorio et al., 2006; Shin et al., 2020). Therefore, appropriate interventions can be devised using the KR-POES to measure the outcome expectations of communication about sex according to the gender of Korean parents.

Moreover, when planning future sex education programs for parents, information on practical communication skills should be included along with the relevant knowledge. Since parental sex education can have a positive effect on enhancing parental satisfaction and outcome expectancy, as well as parents’ relationships with their children (Shin et al., 2020), it is suggested that the effectiveness of education be measured using the KR-POES. If effective education is provided based on the results of the KR-POES, parents’ satisfaction will increase and children’s sexual anxiety will decrease (Cho and Moon, 2016), leading to a positive effect on satisfaction with sex and sexual health communication.

## 5. Conclusion

This study confirmed the reliability and validity of the KR-POES, which comprises of five factors and 22 items, for the evaluation of the outcome expectancy of communication about sex between parents and their children. This culturally-sensitive instrument is a five-point Likert scale, with scores ranging from 22 to 110; higher scores indicate more positive outcome expectancies. The reliability and validity of the KR-POES were verified, which indicates its applicability to Korean parents. Through this instrument, it is possible to measure the outcome expectancy of parents’ sex communication with elementary school children. The instrument can also help parents prepare for helpful communication about sex with their children and for sexual education. Ultimately, this can have a positive effect on children’s sexual health and parental satisfaction in Korea.

## 6. Limitations and implications

The development and validation of the Korean version of the KR-POES for measuring the sexual communication abilities of parents of elementary school students in South Korea can have significant implications for improving sexual well-being among young people. By providing parents with a reliable and valid tool to assess their outcome expectancies regarding communication about sex, the KR-POES empowers parents to actively engage in discussions about sexual and reproductive health with their children.

Sex education at home plays a crucial role in shaping young people’s understanding of sexuality, relationships, and responsible sexual behavior. When parents are equipped with a standardized instrument like the KR-POES, they gain insights into their own beliefs, attitudes, and expectations regarding sex communication. This self-awareness allows them to identify potential barriers to discussing sensitive topics and allows them to prepare for these conversations.

The KR-POES can serve as a guide for parents to initiate and navigate discussions about sex with their children in a supportive and informed manner. By addressing their own outcome expectancies and understanding the potential benefits of open communication, parents can create a safe and non-judgmental environment where their children feel comfortable seeking accurate information and guidance.

Improved sexual well-being for young people can result from various factors that are facilitated using the KR-POES. Firstly, open and honest communication between parents and children promotes the acquisition of accurate knowledge on sexual health, contraception, and STIs. This knowledge equips the youth to make informed decisions, protect themselves, and engage in responsible sexual behaviors when the time comes.

Secondly, discussions about sex within the family foster positive attitudes and values regarding sexuality. By openly addressing topics such as consent, mutual respect, and healthy relationships, parents can instill important values that contribute to the development of healthy sexual behaviors and attitudes later on in life.

Thirdly, the KR-POES can help parents identify and overcome their own discomfort, fears, or misconceptions about discussing sex. By addressing these barriers, parents become more confident and competent in providing accurate information and guidance, reducing the likelihood of misinformation or harmful beliefs being passed onto their children.

Overall, the availability of a reliable and valid instrument like the KR-POES supports parents in actively engaging in discussions about sex and contributes to a positive and healthy climate for sexual activity among young people. By empowering parents to fulfill their role as primary sexuality educators, the KR-POES can have a positive impact on children’s sexual health, decision-making, and overall well-being, while also promoting parental satisfaction in their ability to address this important aspect of their child’s development.

## Data availability statement

The raw data supporting the conclusions of this article will be made available by the authors, without undue reservation.

## Ethics statement

The studies involving human participants were reviewed and approved by the institutional review board of H University in South Korea (HIRB-2022-045). Participants provided informed consent to participate in this study prior to the online survey.

## Author contributions

JL and YK: conception and design of this study, provision of study materials or participants, and critically reviewing the manuscript and supervising the entire study process. JL: performing statistical analyses. JL, YK, and RR: drafting the manuscript. All authors contributed to the article and approved the submitted version.

## Funding

This research was supported by the Hallym University Research Fund, 2022 (HRF-202209-003).

## Conflict of interest

The authors declare that the research was conducted in the absence of any commercial or financial relationships that could be construed as a potential conflict of interest.

## Publisher’s note

All claims expressed in this article are solely those of the authors and do not necessarily represent those of their affiliated organizations, or those of the publisher, the editors and the reviewers. Any product that may be evaluated in this article, or claim that may be made by its manufacturer, is not guaranteed or endorsed by the publisher.

## Abbreviations

POES: parenting outcome expectancy scale
STDs: sexually transmitted diseases
HIV: human immunodeficiency virus
BPSES: brief parenting self-efficacy scale
PACS: parent-adolescent communication scale
KR-POES: Korean version of the POES
EFA: exploratory factor analysis
KMO: Kaiser-Meyer-Olkin
CFA: confirmatory factor analysis
